# Latent class analysis of dyadic psychosocial adaptation in young and middle-aged couples with liver cirrhosis

**DOI:** 10.3389/fpsyg.2026.1798196

**Published:** 2026-06-10

**Authors:** Xue Wang, Shuang Zhang, Huiying An

**Affiliations:** Henan Provincial People’s Hospital, People’s Hospital of Zhengzhou University, Zhengzhou, Henan, China

**Keywords:** dyadic adaptation, family health, financial toxicity, latent profile analysis, liver cirrhosis, psychosocial adjustment, resilience

## Abstract

**Objective:**

This study aimed to identify distinct dyadic psychosocial adaptation profiles among patients with liver cirrhosis and their spouses, and to examine clinical and psychosocial factors associated with profile membership.

**Methods:**

In this cross-sectional study conducted in a tertiary hospital in Zhengzhou, China, between February 2023 and August 2024, 321 patient–spouse dyads completed validated measures including the Psychosocial Adjustment to Illness Scale (PAIS-SR), the 10-item Connor-Davidson Resilience Scale (CD-RISC-10), the 10-item Family Health Scale-Short Form (FHS-SF), and the 11-item Comprehensive Score for Financial Toxicity (COST). Latent profile analysis (LPA) was conducted to derive dyadic adaptation profiles, and multinomial logistic regression was conducted to examine the predictors of maladaptive profiles.

**Results:**

Five distinct dyadic profiles were identified—dyadic maladjustment (*n* = 49, 15.27%), patient-predominant maladjustment (*n* = 59, 18.38%), spouse-predominant maladjustment (*n* = 75, 23.37%), pseudo-synergistic adaptation (*n* = 95, 29.60%), and dyadic positive adaptation (*n* = 43, 13.40%). Lower resilience, poorer family health, greater financial toxicity, and lower patient education were independently associated with the membership in maladaptive profiles (all *p*-values <0.05). These profiles exhibited significant differences in clinical severity and psychosocial burden.

**Conclusion:**

Dyadic psychosocial adaptation in liver cirrhosis is heterogeneous and shaped by the interplay of personal, familial, and socioeconomic factors rather than clinical severity alone. Early dyadic screening for resilience, family functioning, health literacy, and financial strain may guide precision-targeted, couple-centered interventions to enhance adaptation and quality of life for both patients and their spouses.

## 1 Introduction

Chronic liver disease and cirrhosis impose an immense and growing health burden worldwide, particularly among working-age adults. Liver disease accounts for approximately 2 million deaths annually (≈4% of global mortality) (Devarbhavi et al., 2023), with cirrhosis complications responsible for the majority of fatalities. In China, the 2019 Global Burden of Disease Study estimated that cirrhosis resulted in 560.4 age-standardized disability-adjusted life years (DALYs) per 100,000 population—among the highest national rates globally—with individuals aged 30–50 years contributing to 30–50% of all cirrhosis-related deaths or DALYs (Liu et al., 2024).

Although hepatitis B virus (HBV) historically accounted for the majority of cirrhosis cases in China, its proportion has declined with widespread vaccination and antiviral therapy from 82.4% (2001–2005) to 74.2% (2016–2020) (Wang et al., 2023). Non-alcoholic fatty liver disease (NAFLD) has emerged as the predominant etiology, accompanied by a rising prevalence of alcoholic cirrhosis. National surveys indicate that the prevalence of NAFLD increased from 25.4% (2008–2010) to 32.3% (2015–2018) among Chinese adults (Wong et al., 2023), paralleling the rapid growth of obesity and metabolic syndrome. Where there is a gradual shift toward older age groups, working-age individuals (20–59 years) still represent a substantial portion of cirrhosis-related disability and mortality, with middle-aged men being particularly vulnerable (Duo et al., 2025).

Cirrhosis imposes severe economic consequences: direct inpatient costs for HBV-related cirrhosis exceed CNY 16,800 per admission, while indirect income losses add nearly CNY 4,800. For many households, medical expenses can account for almost 80% of total expenditures (Ma et al., 2017). These economic pressures compound patients’ psychosocial challenges, including diminished work capacity, disrupted family roles, and limited social participation.

Psychosocial adaptation refers to the psychological and social adjustment to chronic illness by both patients and their families (Derogatis, 1986). Poor adaptation in cirrhosis is common and may manifest as anxiety, depression, or social withdrawal (Jafree et al., 2024; Valery and Bernardes, 2021). Meta-analyses report that the prevalence of depression among cirrhosis patients can be as high as 65%, while the prevalence of anxiety is 37% (Wang et al., 2012; Volk, 2020). Additionally, caregivers—predominantly spouses—also experience high levels of psychological distress, reduced quality of life, and a heavy caregiving burden (Shrestha et al., 2020). Importantly, psychosocial strain in one partner can exacerbate distress in the other, creating a mutually reinforcing cycle (Ufere et al., 2025). Dyadic coping theory emphasizes that couples manage illness through mutual support, communication, and coordinated coping strategies, which are strong predictors of both partners’ psychosocial adjustment (Weitkamp et al., 2021; Li et al., 2022).

The majority of previous studies have examined patients and spouses separately, using aggregate scores that overlook within-couple heterogeneity (Sun, 2023; Pegum et al., 2015). In contrast, person-centered statistical approaches, such as latent profile analysis (LPA), allow the identification of unobserved subgroups sharing similar patterns across multiple psychosocial domains. In chronic illness populations, dyadic LPAs have uncovered distinct adaptation typologies that are meaningfully associated with demographic, relational, and contextual factors (Novak et al., 2021; Wei et al., 2024; Wang et al., 2023). However, to date, no study has applied LPA to examine dyadic psychosocial adaptation in couples coping with liver cirrhosis.

Guided by the Transactional Social-Ecological theory, psychosocial adaptation is shaped by personal characteristics (e.g., resilience), familial characteristics (e.g., family health), and environmental characteristics (e.g., financial well-being) (Kuldas and Foody, 2022; Saltzman and Hansel, 2024). Lower resilience has been associated with greater frailty and poorer health outcomes in patients with cirrhosis (Wong et al., 2021). Poor family functioning may limit coping resources (Mao et al., 2021), while higher financial toxicity—defined as the economic distress and burden caused by illness—is linked to poorer quality of life and greater psychological distress (Ufere et al., 2022). Given that the above-mentioned personal (resilience), familial (family health), and environmental characteristics (financial toxicity) play important roles in predicting psychosocial adaptation, it is helpful to examine the independent effects of these factors on distinct dyadic psychosocial adaptation profiles.

Understanding dyadic psychosocial adaptation patterns in couples facing liver cirrhosis has both theoretical and practical significance. Theoretically, these patterns extend dyadic coping and transactional stress frameworks into the context of chronic liver disease, offering a nuanced view of how patient–spouse interactions can shape adaptation outcomes. Practically, identifying distinct adaptation profiles enables clinicians to recognize vulnerable dyads who may require targeted psychosocial interventions, tailored support for both partners, and resource allocation that addresses personal, familial, and financial determinants. These insights may inform the design of couple-centered care models and integrated support programs, ultimately aiming to improve psychological well-being, relational functioning, and quality of life for both members of the dyad.

Accordingly, this study aimed to:

1. Identify distinct dyadic psychosocial adaptation profiles among young and middle-aged couples coping with liver cirrhosis;
2. Compare profiles with respect to demographic, clinical, personal (resilience), familial (family health), and environmental (financial toxicity) factors;
3. Examine the combined effects of these predictors on dyadic adaptation patterns.

## 2 Methods

### 2.1 Study design and settings

This single-center study was conducted in the Department of Gastroenterology at a tertiary hospital in Zhengzhou, Henan Province, China. The hospital included a diverse patient population from both urban and rural areas across central China. Participants were recruited from February 2023 to August 2024, primarily from inpatient wards managing liver disease patients.

### 2.2 Participants and procedures

The inclusion criteria were as follows: (a) patients with a confirmed diagnosis of cirrhosis, established either by liver biopsy histopathology or by compatible clinical, laboratory, and imaging findings [such as nodular liver contour and/or signs of portal hypertension on ultrasound, computed tomography (CT), or magnetic resonance imaging (MRI)] as assessed by at least two hepatologists; (b) spouse as the primary caregiver; (c) both patients and spouses aged between 18 and 60 years; and (d) both partners free from psychiatric disorders, capable of effective communication, and able to provide informed consent. The exclusion criteria were as follows: (a) patients with severe chronic conditions unrelated to cirrhosis, such as cerebrovascular accidents (CVA), dementia, chronic kidney disease (CKD), or any psychiatric disorders; and (b) patients with overt hepatic encephalopathy (HE), as cognitive impairment may affect the validity of self-reported questionnaire data. The sample size was determined based on recommendations for LPA and requirements for multivariable regression. Simulation studies and prior literature suggest that a minimum sample size of approximately 300 is required to ensure stable class enumeration and reliable parameter estimation (Tein et al., 2013). The final sample of this study included 321 dyads, which aligns with this recommendation.

This cross-sectional study was approved by the Institutional Ethics Committee of Henan Provincial People’s Hospital (No. 2022-141) and employed a convenience sampling method. Before the study, nurses received comprehensive training on research methods and data collection tools. After screening for eligibility based on inclusion criteria, nurses provided potential participants with written information about the study and obtained their informed consent. Data collection was conducted using structured questionnaires. Patients and their spouses completed questionnaires separately in paper-and-pencil format by trained research nurses in a quiet, private setting. No financial or material incentives were offered. A total of 330 eligible dyads consented to participate and received the questionnaires. All returned questionnaires were reviewed on-site for completeness. Minor omissions were promptly corrected, but nine dyads were excluded due to substantial incomplete data (such as an entire scale left blank), yielding a final analytic sample of 321 dyads with complete data. The number of dyads who declined participation was not formally recorded.

### 2.3 Measurement

All multi-item scales were scored and coded according to the standard procedures described in their original development and validation studies. For the COST scale, reverse coding was applied to relevant items when required. Completed questionnaires were checked for completeness on-site by trained research nurses; any minor omissions were promptly addressed. Consequently, no missing data remained in the final analytic sample. Internal consistency of all scales in the present sample was assessed using Cronbach’s alpha; with values >0.80 considered indicative of good reliability (Nunnally and Bernstein, 1994). The full versions of all questionnaires are provided in Supplementary File 1.

Patients completed items on sociodemographic and cirrhosis-related variables, including age, gender, place of residence, education level, family monthly income per capita, proportion of medical reimbursement, number of hospitalizations, disease course, liver function classification, and type of liver cirrhosis. Spouses completed sociodemographic variables such as age, gender, education level, occupation type, chronic disease, and employment status. In addition, the following four instruments were administered.

#### 2.3.1 Psychosocial adjustment

The 44-item Self-Report Psychosocial Adjustment to Illness Scale (PAIS-SR) was used to assess patients’ psychosocial adjustment. The scale (Yao, 2013; MAPI Research Trust, 2026) comprises seven domains: work ability (6 items), healthcare orientation (7 items), family relationships (7 items), sexual life (6 items), communication (5 items), recreational activities (6 items), and psychological status (7 items). Each PAIS-SR item is rated on a 4-point (0–3) scale of adjustment, with higher ratings indicating poorer adjustment. On the PAIS-SR, item direction alternates to reduce position response biases. Total scores range from 0 to 132, with higher scores indicating poorer psychosocial adjustment. A parallel caregiver version, identical in item content and structure but prefaced with “As a caregiver, I…”, was administered to spouses to assess their own psychosocial adjustment. The PAIS-SR has demonstrated good reliability and validity across multiple chronic illness populations, with evidence of predictive, convergent, and construct validity from factor analytic studies. Internal consistency estimates for the PAIS-SR ranged from 0.50 to 0.86 across subscales (Rodrigue et al., 2000). In the present sample, Cronbach’s *α* was 0.915 for the patient version and 0.872 for the spouse version, both indicating excellent to good internal consistency.

#### 2.3.2 Resilience

The 10-item Connor-Davidson Resilience Scale (CD-RISC-10) (Zhang et al., 2018) was used to assess resilience. Each item was rated on a 5-point Likert scale ranging from “Not true at all” = 0 to “True nearly always” = 4, with higher scores indicating greater resilience. The CD-RISC-10 has a single-factor structure and demonstrated excellent psychometric properties in Chinese populations, with Cronbach’s *α* = > 0.93, McDonald’s *ω* = > 0.93, and test–retest reliability (ICC = 0.88) (Tu et al., 2023). In the present sample, Cronbach’s *α* was 0.832 for the patient version and 0.815 for the spouse version, both indicating good internal consistency.

#### 2.3.3 Family health

The 10-item Family Health Scale-Short Form (FHS-SF) (Wang et al., 2022) was used to assess family health. The scale operationalizes family health as a multidimensional construct comprising four dimensions: (1) family social/emotional health processes, (2) family healthy lifestyle, (3) family health resources, and (4) family external social supports. Participants rated each item on a 5-point Likert scale ranging from “Strongly disagree” = 1 to “Strongly agree” = 5. A total score was calculated as the sum of all 10 items (range: 10–50), with higher scores indicating better family health. The Chinese version of the FHS-SF was validated in a national sample of 8,912 residents (Wang et al., 2022), demonstrating good reliability and validity [total scale Cronbach’s *α* = 0.83, subscale α range 0.70–0.90; test–retest reliability = 0.75; CFA model fit: goodness of fit index (GFI) = 0.98, normed fit index (NFI) = 0.97, relative fit index (RFI) = 0.95, and root mean square error of approximation (RMSEA) = 0.07]. In the present sample of spouses, Cronbach’s *α* was 0.792, indicating acceptable internal consistency.

#### 2.3.4 Financial toxicity

The Chinese version of the 11-item Comprehensive Score for Financial Toxicity (COST) (Yu et al., 2017) was used to assess patients’ financial toxicity. Financial toxicity refers to the objective economic burden and subjective financial distress (such as out-of-pocket costs, income loss, and financial worry) experienced by patients and families as a consequence of medical illness and its treatment. Each item was rated on a 5-point Likert scale from “Not at all” = 0 to “Very much” = 4. Following the standard scoring protocol, certain items were reverse-coded prior to calculate the total score. Total scores range from 0 to 44, with higher scores reflecting lower financial toxicity (such as better financial well-being). The COST has demonstrated good internal consistency (Cronbach’s α = 0.89) and predictive validity in chronic disease populations, with lower financial toxicity associated with better physical and mental health-related quality of life (Pavela et al., 2021). In the present sample, Cronbach’s α was 0.856 among patients, indicating good internal consistency, which is consistent with published values in chronic disease populations.

### 2.4 Statistical methods

LPA was conducted using Mplus version 8.7 (Muthén and Muthén, 1998–2017) to identify distinct psychosocial adaptation profiles among couples based on seven dimensions of the PAIS-SR, including healthcare orientation, vocational environment, domestic relationships, sexual relationships, extended relationships, social environment, and psychological distress. Models specifying 1–6 profiles were estimated. To avoid local maxima, each model was tested using 1,000 random initial stage starts and 250 final stage optimizations. Model fit was assessed using the Akaike Information Criterion (AIC), Bayesian Information Criterion (BIC), and sample-size-adjusted BIC (aBIC), with lower values indicating better model fit (Nylund-Gibson and Choi, 2018). Entropy values (ranging from 0 to 1) were used to evaluate classification accuracy, with values closer to 1 indicating more precise classification. The Lo–Mendell–Rubin Adjusted Likelihood Ratio Test (LMRT) and the Bootstrap Likelihood Ratio Test (BLRT) were performed to compare models with *k* versus *k* − 1 profiles; a *p*-value of <0.05 indicated that the model with *k* profiles significantly improved the model fit. The final number of latent profiles was determined based on a combination of statistical indices, theoretical interpretability, and parsimony. No formal alpha error correction (such as Bonferroni adjustment) was applied to the sequential LMRT and BLRT comparisons, consistent with the prevailing methodological norms in LPA literature (Spurk et al., 2020). This reflects the nature of these comparisons as nested, sequential model tests rather than a family of independent parallel tests, as well as the multi-criterion approach to class enumeration, which relies on the convergence of multiple fit indices alongside theoretical interpretability rather than any single significance test.

Descriptive statistics were conducted using SPSS version 26.0 to summarize participants’ sociodemographic and clinical characteristics. Categorical variables were reported as frequencies and percentages, while non-normally distributed continuous variables were presented as medians and interquartile ranges (P25–P75). Following latent profile identification, group differences in demographic characteristics, psychological resilience, family health, and financial toxicity were examined. Categorical variables were compared using the Chi-squared test or Fisher’s exact test. Continuous variables were compared using the Kruskal–Wallis *H* test. To identify independent predictors of profile membership, multinomial logistic regression was performed with latent profile membership as the dependent variable and demographic and psychosocial variables (such as age, gender, resilience, family health, and financial toxicity) as predictors. A *p*-value of <0.05 was considered statistically significant.

## 3 Results

### 3.1 Sociodemographic characteristics and psychosocial adjustment of participants

A total of 330 questionnaires were distributed, and 321 valid responses were collected, yielding a valid response rate of 97.27%.

Patients with cirrhosis ranged in age from 29 to 60 years (mean ± SD: 45.83 ± 5.35). The majority were men (*n* = 214, 66.7%). Regarding education, 31 (9.7%) had completed elementary school, 79 (24.6%) junior high school, 116 (36.1%) high school or technical secondary school, and 95 (29.6%) university or college education. The mean psychosocial adjustment score was 56.98 ± 19.42 (range: 27 ~ 103), the mean resilience score was 28.05 ± 4.15 (range: 17 ~ 40), and the mean financial toxicity score was 25.03 ± 4.65 (range: 7 ~ 39).

Spouses ranged in age from 32 to 60 years (mean ± SD: 46.12 ± 5.87). The majority were women (*n* = 214, 66.7%). Regarding education, 40 (12.5%) had completed elementary school, 96 (29.9%) junior high school, 119 (37.1%) high school or technical secondary school, and 66 (20.6%) university or college education. The mean psychosocial adjustment score was 53.75 ± 20.20 (range: 24 ~ 96), the mean resilience score was 27.93 ± 4.72 (range: 15 ~ 40), and the mean family health score was 29.65 ± 4.79 (range: 18 ~ 42). Additional sociodemographic and clinical information for both patients and spouses is presented in Table 1.

**Table 1:** Univariate analysis of demographic and clinical characteristics across latent psychosocial adaptation profiles among young and middle-aged patients with liver cirrhosis and their spouses (*n* = 321).

Characteristics	Mean ± SD or *n* (%)	Group 1 (*n* = 49)	Group 2 (*n* = 59)	Group 3 (*n* = 75)	Group 4 (*n* = 95)	Group 5 (*n* = 43)	t/*F* value	*p* value
Patients								
Age (years)	45.83 ± 5.35	43.43 ± 5.079	46.93 ± 4.891	45.28 ± 5.218	46.74 ± 5.46	45.98 ± 5.505	4.113^1^	0.003
Sex							9.146^2^	0.058
Male	214 (66.7%)	38 (77.6%)	33 (55.9%)	48 (64%)	61 (64.2%)	34 (79.1%)		
Female	107 (33.3%)	11 (22.4%)	26 (44.1%)	27 (36%)	34 (35.8%)	9 (20.9%)		
Place of residence							5.366^2^	0.252
Urban area	133 (41.4%)	19 (38.8%)	24 (40.7%)	39 (52%)	33 (34.7%)	18 (41.9%)		
Rural area	188 (58.6%)	30 (61.2%)	35 (59.3%)	36 (48%)	62 (65.3%)	25 (58.1%)		
Education							22.388^3^	**0.033**
Elementary school	31 (9.7%)	3 (6.1%)	12 (20.3%)	8 (10.7%)	7 (7.4%)	1 (2.3%)		
Junior high school	79 (24.6%)	11 (22.4%)	21 (35.6%)	17 (22.7%)	22 (23.2%)	8 (18.6%)		
High school/Technical secondary school	116 (36.1%)	18 (36.7%)	14 (23.7%)	28 (37.3%)	40 (42.1%)	16 (37.2%)		
University or college education	95 (29.6%)	17 (34.7%)	12 (20.3%)	22 (29.3%)	26 (27.4%)	18 (41.9%)		
Average household income per capita (RMB)							35.345^3^	**<0.001**
<3,000	46 (14.3%)	9 (18.4%)	13 (22%)	11 (14.7%)	12 (12.6%)	1 (2.3%)		
3,000–5,000	130 (40.5%)	31 (63.3%)	21 (35.6%)	33 (44%)	33 (34.7%)	12 (27.9%)		
5,000–10,000	114 (35.5%)	7 (14.3%)	17 (28.8%)	25 (33.3%)	39 (41.1%)	26 (60.5%)		
>10,000	31 (9.7%)	2 (4.1%)	8 (13.6%)	6 (8%)	11 (11.6%)	4 (9.3%)		
Medical insurance reimbursement rate							19.633^3^	0.074
Fully self-paid	18 (5.6%)	3 (6.1%)	3 (5.1%)	7 (9.3%)	2 (2.1%)	3 (7%)		
<25% reimbursed	49 (15.3%)	13 (26.5%)	10 (16.9%)	8 (10.7%)	8 (8.4%)	10 (23.3%)		
25–<50% reimbursed	181 (56.4%)	25 (51%)	34 (57.6%)	46 (61.3%)	56 (58.9%)	20 (46.5%)		
50–<75% reimbursed	73 (22.7%)	8 (16.3%)	12 (20.3%)	14 (18.7%)	29 (30.5%)	10 (23.3%)		
≥75% reimbursed	16 (5%)	3 (6.1%)	3 (5.1%)	7 (9.3%)	2 (2.1%)	1 (2.3%)		
Number of hospitalizations							2.187^2^	0.975
1	134 (41.7%)	21 (42.9%)	23 (39%)	35 (46.7%)	37 (38.9%)	18 (41.9%)		
2–3	89 (27.7%)	15 (30.6%)	16 (27.1%)	19 (25.3%)	26 (27.4%)	13 (30.2%)		
≥4	98 (30.5%)	13 (26.5%)	20 (33.9%)	21 (28%)	32 (33.7%)	12 (27.9%)		
Time since cirrhosis diagnosis (years)								
<1	60 (18.7%)	15 (30.6%)	10 (16.9%)	13 (17.3%)	17 (17.9%)	5 (11.6%)	17.612^2^	0.024
1–5	158 (49.2%)	22 (44.9%)	22 (37.3%)	46 (61.3%)	48 (50.5%)	20 (46.5%)		
>5	103 (32.1%)	12 (24.5%)	27 (45.8%)	16 (21.3%)	30 (31.6%)	18 (41.9%)		
Child-Pugh classification							23.451^3^	**0.003**
Child-Pugh A	145 (45.2%)	26 (53.1%)	30 (50.8%)	25 (33.3%)	33 (34.7%)	31 (72.1%)		
Child-Pugh B	134 (41.7%)	17 (34.7%)	22 (37.3%)	37 (49.3%)	48 (50.5%)	10 (23.3%)		
Child-Pugh C	42 (13.1%)	6 (12.2%)	7 (11.9%)	13 (17.3%)	14 (14.7%)	2 (4.7%)		
Etiology of cirrhosis							4.720^3^	0.787
Alcohol	209 (65.1%)	34 (69.4%)	40 (67.8%)	45 (60%)	61 (64.2%)	29 (67.4%)		
Hepatitis	74 (23.1%)	11 (22.4%)	12 (20.3%)	21 (28%)	19 (20%)	11 (25.6%)		
Others	38 (11.8%)	4 (8.2%)	7 (11.9%)	9 (12%)	15 (15.8%)	3 (7%)		
Ascites							2.194^2^	0.700
No	229 (71.3%)	35 (71.4%)	42 (71.2%)	49 (65.3%)	70 (73.7%)	33 (76.7%)		
Yes	92 (28.7%)	14 (28.6%)	17 (28.8%)	26 (34.7%)	25 (26.3%)	10 (23.3%)		
Has gastrointestinal bleeding occurred							11.448^2^	**0.022**
No	211 (65.7%)	36 (73.5%)	36 (61%)	39 (52%)	67 (70.5%)	33 (76.7%)		
Yes	110 (34.3%)	13 (26.5%)	23 (39%)	36 (48%)	28 (29.5%)	10 (23.3%)		
Family structure							6.707^2^	0.569
Nuclear family	166 (51.7%)	26 (53.1%)	34 (57.6%)	36 (48%)	46 (48.4%)	24 (55.8%)		
Stem family	81 (25.2%)	8 (16.3%)	14 (23.7%)	20 (26.7%)	30 (31.6%)	9 (20.9%)		
Couple-only family	74 (23.1%)	15 (30.6%)	11 (18.6%)	19 (25.3%)	19 (20%)	10 (23.3%)		
Resilience	28.05 ± 4.151	26.96 ± 4.041	26.25 ± 3.026	28.73 ± 4.691	28.89 ± 4.145	28.72 ± 3.731	5.694^1^	<0.001
Financial toxicity	25.03 ± 4.645	23.16 ± 4.317	25.24 ± 4.588	25.37 ± 4.567	25.11 ± 4.83	26.14 ± 4.411	2.791^1^	0.027

Spouses								
Age (years)	46.12 ± 5.87	43.98 ± 5.532	47.47 ± 5.338	44.99 ± 5.617	47.32 ± 6.101	46.07 ± 5.982	0.755^1^	0.812
Sex	206 (64.2%)	32 (65.3%)	36 (61%)	51 (68%)	57 (60%)	30 (69.8%)	0.080^2^	0.724
Male	107 (33.3%)	17 (34.7%)	23 (39%)	24 (32%)	30 (31.6%)	13 (30.2%)		
Female	214 (66.7%)	32 (65.3%)	36 (61%)	51 (68%)	65 (59.4%)	30 (69.8%)		
Education							30.789^3^	**0.002**
Elementary school	40 (12.5%)	6 (12.2%)	7 (11.9%)	14 (18.7%)	11 (11.6%)	2 (4.7%)		
Junior high school	96 (29.9%)	14 (28.6%)	15 (25.4%)	36 (48%)	20 (21.1%)	11 (25.6%)		
High school/Technical secondary school	119 (37.1%)	20 (40.8%)	27 (45.8%)	18 (24%)	37 (38.9%)	17 (39.5%)		
University or college education	66 (20.6%)	9 (18.4%)	10 (16.9%)	7 (9.3%)	27 (28.4%)	13 (30.2%)		
Occupational type							10.198^2^	0.599
Unemployed	37 (11.5%)	7 (14.3%)	8 (13.6%)	10 (13.3%)	6 (6.3%)	6 (14%)		
Migrant worker	63 (19.6%)	9 (18.4%)	13 (22%)	12 (16%)	21 (22.1%)	8 (18.6%)		
Company employee/Teacher	139 (43.3%)	20 (40.8%)	19 (32.2%)	37 (49.3%)	47 (49.5%)	16 (37.2%)		
Professor/Manager	82 (25.5%)	13 (26.5%)	19 (32.2%)	16 (21.3%)	21 (22.1%)	13 (30.2%)		
Spouse with a chronic disease							11.661^2^	**0.020**
Yes	17 (34.7%)	24 (40.7%)	21 (28%)	42 (44.2%)	25 (58.1%)	129 (40.2%)		
No	32 (65.3%)	35 (59.3%)	54 (72%)	53 (55.8%)	18 (41.9%)	192 (59.8%)		
Currently employed							15.049^2^	**0.005**
Yes	114 (35.5%)	15 (30.6%)	19 (32.2%)	16 (21.3%)	46 (48.4%)	18 (41.9%)		
No	207 (64.5%)	34 (69.4%)	40 (67.8%)	59 (78.7%)	49 (51.6%)	25 (58.1%)		
Resilience	27.93 ± 4.716	27.49 ± 4.316	28 ± 4.72	26.73 ± 4.557	28.24 ± 4.672	29.74 ± 5.062	3.090^1^	0.016
Family health	29.65 ± 4.787	29.65 ± 5.426	30.08 ± 4.966	29.37 ± 4.289	28.78 ± 4.52	31.49 ± 4.813	2.608^1^	0.036

^1^*F* value; ^2^*χ*^2^; ^3^Fisher’s exact test.

### 3.2 Latent profile analysis of dyadic psychosocial adjustment

**Figure 1:**
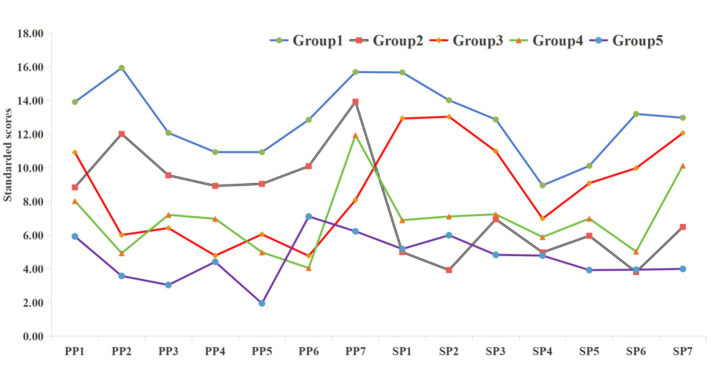
Dyadic psychosocial adaptation profiles in young and middle-aged couples with liver cirrhosis. PP1 represents work ability (patients), PP2 represents health-care orientation (patients), PP3 represents family relationships (patients), PP4 represents sexual life (patients), PP5 represents communication (patients), PP6 represents recreational activities (patients), and PP7 represents psychological status (patients); SP1 represents work ability (spouses), SP2 represents health-care orientation (spouses), SP3 represents family relationships (spouses), SP4 represents sexual life (spouses), SP5 represents communication (spouses), SP6 represents recreational activities (spouses), and SP7 represents psychological status (spouses). Group 1: dyadic maladjustment profile; Group 2: patient maladjustment profile; Group 3: spouse maladjustment profile; Group 4: pseudo-synergic adaptation profile; Group 5: dyadic positive adjustment profile.

LPA was performed using the seven subscales of the PAIS-SR to explore dyadic psychosocial adjustment among patients and their spouses. Models with 1–6 profiles were tested (see Table 2). Fit indices including AIC, BIC, and aBIC progressively decreased with the addition of profiles, and all entropy values were above 0.8. The five-profile model demonstrated the highest entropy and yielded statistically significant LMRT and BLRT results (*p* < 0.001), suggesting optimal model fit. Therefore, the five-profile solution was selected based on model fit, parsimony, and interpretability. Mean scores of the five latent profiles across the seven PAIS-SR subdomains are illustrated in Figure 1.

**Table 2:** Model fit indices for latent profile analysis of psychosocial adaptation among young and middle-aged patients with liver cirrhosis and their spouses (*n* = 321).

Profile	AIC	BIC	aBIC	Entorpy	*p* (LMRT)	*p* (BLRT)	Category probability(%)
1	23224.151	23329.751	23240.939	0.80			1
2	20784.438	20946.610	20810.221	0.901	<0.001	<0.001	0.61371/0.38629
3	19463.203	19681.947	19497.980	0.913	<0.001	<0.001	0.23364/0.61371/0.15265
4	18152.006	18427.321	18195.776	0.956	<0.001	<0.001	0.42936/0.18432/0.23367/0.15265
5	16812.747	17144.634	16865.511	0.960	<0.001	<0.001	0.13396/0.18380/0.29595/0.15265/0.23365
6	16805.692	17194.150	16867.450	0.832	0.385	<0.001	0.13396/0.00935/0.23364/0.29595/0.17445/0.15265

In Group 1 (*n* = 49, 15.27%), labeled as dyadic maladjustment profile, both patient and spouse showed high levels of maladjustment across all domains. Group 2 (*n* = 59, 18.38%), labeled as patient maladjustment profile, patients’ domain scores approximated those observed in Group 1 (dyadic maladjustment), particularly in the domains of work ability, psychological distress, and health-care orientation, whereas spouses’ scores remained markedly lower, approaching the levels seen in the better-adapted groups. This pattern suggests that patients in these dyads bear the predominant psychosocial burden, which may be driven by illness-related role disruption and identity threat, while spouses maintain relatively preserved adaptive capacity. Group 3 (*n* = 75, 23.37%), labeled as spouse maladjustment profile, spouses demonstrated notably higher maladjustment than patients across all domains. Spouses’ domain scores were elevated to a level comparable to those of spouses in Group 1, with the greatest burden in psychological distress, family relationships, and domestic environment. In contrast, patients’ scores were modestly elevated but remained substantially below those of their spouses. The couple-level discrepancy was most prominent in the health-care orientation and psychological distress domains, which likely reflect the spouses’ intense caregiving involvement and emotional strain. In Group 4 (*n* = 95, 29.60%), labeled as pseudo-synergic adaptation profile, both partners exhibit similar, moderate-level profiles across domains, superficially resembling a coordinated coping pattern. However, this is termed “pseudo-synergistic” because the psychological distress dimension remains relatively elevated, suggesting concealed distress beneath the surface; indicating that the apparent synergy may rest on a fragile foundation with limited deep coping resources. Such a “surface-level alignment” may be easily overlooked in clinical practice. Group 5 (*n* = 43, 13.40%), labeled as dyadic positive adjustment profile, both partners showed the lowest maladjustment scores across all domains.

### 3.3 Univariate analysis of dyadic psychosocial adjustment profiles

Univariate analyses were conducted to examine differences in demographic and psychosocial variables across the five latent profiles. Results are summarized in Table 1.

### 3.4 Multinomial logistic regression analysis of latent profiles

To identify independent predictors of profile membership, multinomial logistic regression was conducted with the five latent profiles as the dependent variable and the dyadic positive adaptation profile (Group 5) as the reference category. All sociodemographic and clinical variables that were statistically significant (*p* < 0.05) in univariate analyses were simultaneously entered into a single model (see Table 3).

**Table 3:** Multinomial logistic regression predicting profile membership (reference: Group 5, dyadic positive adaptation).

Characteristics	Group 1 (dyadic maladjustment)	Group 2 (patient maladjustment)	Group 3 (spouse maladjustment)	Group 4 (pseudo-synergistic)
	OR (95% CI), *p*	OR (95% CI), *p*	OR (95% CI), *p*	OR (95% CI), *p*
Patient age	**0.87 (0.79–0.97), 0.010**	1.03 (0.94–1.13), 0.545	1.00 (0.91–1.09), 0.973	1.04 (0.96–1.13), 0.329
Patient education				
Junior high school	2.11 (0.55–8.06), 0.276	**4.32 (1.23–15.18), 0.023**	1.98 (0.56–6.96), 0.287	2.45 (0.78–7.69), 0.126
Elementary school	3.00 (0.20–43.94), 0.423	**21.39 (1.90–240.29), 0.013**	11.23 (0.95–133.03), 0.055	8.88 (0.80–98.20), 0.075
Spouse education				
Junior high school	1.38 (0.45–4.28), 0.577	1.09 (0.36–3.27), 0.884	**9.35 (2.50–34.98), 0.001**	1.19 (0.39–3.66), 0.766
Elementary school	2.56 (0.62–10.52), 0.193	1.94 (0.50–7.51), 0.337	**19.17 (2.65–138.77), 0.003**	2.86 (0.46–17.89), 0.262
Average household income per capita (RMB)				
3,000–5,000	**10.08 (1.28–79.29), 0.028**	1.03 (0.41–2.60), 0.955	1.15 (0.47–2.84), 0.758	0.89 (0.40–2.00), 0.785
<3,000	**24.50 (1.29–466.71), 0.033**	1.28 (0.35–4.68), 0.710	1.42 (0.40–5.07), 0.590	1.19 (0.36–3.92), 0.771
Time since cirrhosis diagnosis (years)				
1–5	1.67 (0.53–5.30), 0.382	0.77 (0.28–2.16), 0.622	**3.50 (1.24–9.89), 0.018**	1.60 (0.64–3.98), 0.315
<1	**5.51 (1.17–25.94), 0.031**	1.62 (0.37–7.04), 0.523	3.69 (0.84–16.16), 0.083	2.47 (0.63–9.68), 0.196
GI bleeding (No)	1.09 (0.35–3.41), 0.878	0.69 (0.25–1.91), 0.471	0.40 (0.15–1.08), 0.069	1.07 (0.41–2.74), 0.897
Child-Pugh A	0.23 (0.05–1.22), 0.085	0.31 (0.06–1.56), 0.156	**0.21 (0.04–0.97), 0.045**	**0.22 (0.05–0.95), 0.042**
Spouse chronic disease (No)	0.51 (0.18–1.42), 0.195	0.50 (0.19–1.29), 0.151	**0.30 (0.12–0.77), 0.013**	0.51 (0.22–1.19), 0.120
Spouse employment (Yes)	0.87 (0.32–2.37), 0.789	0.93 (0.35–2.46), 0.884	0.65 (0.25–1.72), 0.390	1.25 (0.56–2.82), 0.591
Resilience (patient)	**0.81 (0.71–0.93), 0.003**	**0.84 (0.75–0.95), 0.005**	1.04 (0.92–1.17), 0.559	1.03 (0.92–1.15), 0.614
Resilience (spouse)	0.92 (0.83–1.03), 0.147	0.92 (0.83–1.01), 0.076	**0.84 (0.76–0.93), 0.001**	**0.91 (0.84–1.00), 0.045**
Family health	0.93 (0.84–1.04), 0.223	0.91 (0.82–1.01), 0.073	**0.88 (0.79–0.97), 0.011**	**0.84 (0.76–0.93), <0.001**
Financial toxicity	**0.84 (0.75–0.95), 0.005**	0.96 (0.86–1.06), 0.406	0.98 (0.89–1.09), 0.761	0.96 (0.87–1.06), 0.400

Bold = *p* < 0.05. OR = odds ratio; CI = confidence interval. Group 5 (dyadic positive adaptation) serves as the reference category. All predictors that were significant (*p* < 0.05) in univariate analyses were simultaneously entered into a single multinomial logistic regression model. Non-significant ORs are reported in full for model completeness and consistency with standard multinomial regression reporting practice.

Compared with the dyadic positive adaptation group, membership in the dyadic maladjustment group (Group 1) was independently associated with younger age (OR = 0.87), lower household income (<3,000 RMB: OR = 24.50; 3,000–5,000 RMB: OR = 10.08), disease duration <1 year (OR = 5.51), lower resilience (OR = 0.81), and greater financial toxicity (OR = 0.84). Membership in the patient maladjustment group (Group 2) was independently associated with lower patient education (elementary school: OR = 21.39; junior high school: OR = 4.32) and lower resilience (OR = 0.84). Membership in the spouse maladjustment group (Group 3) was independently associated with disease duration of 1–5 years (OR = 3.50), less severe liver disease (Child-Pugh A: OR = 0.21), lower spouse education (elementary school: OR = 19.17; junior high school: OR = 9.35), absence of spouse chronic disease (OR = 0.30), lower resilience (OR = 0.84), and lower family health (OR = 0.88). Membership in the pseudo-synergistic adaptation group (Group 4) was independently associated with less severe liver disease (Child-Pugh A: OR = 0.22), lower resilience (OR = 0.91), and lower family health (OR = 0.84).

## 4 Discussion

This study identified five distinct dyadic psychosocial adaptation profiles among young and middle-aged couples managing liver cirrhosis. These ranged from dyadic maladjustment (both partners poorly adapting), through patient- or spouse-maladjustment (one partner struggling, the other relatively well), and pseudo-synergistic (moderate mutual coping that may mask underlying distress), to dyadic adjustment (both partners coping well). These findings echo prior work showing that when both partners engage collaboratively in coping, outcomes improve, but when both are impaired, psychological morbidity is severe (Ufere et al., 2025; Weitkamp et al., 2021). Studies of chronic liver disease (CLD) consistently report high rates of anxiety and depression that worsen as symptom burden grows—patterns reflected in the “dyadic maladjustment” group (Jafree et al., 2024). Conversely, oncology studies demonstrate that couples adopting a relational “we” orientation cope better than those coping individually (D’Arrigo-Patrick et al., 2020), consistent with the dyadic adjustment positive profile. Latent profile analyses in cancer populations have revealed multiple coping subgroups, with “high-level” coping couples showing the best outcomes and “low-level” coping couples requiring intensive support (Zhang et al., 2024).

Compared with the dyadic positive adaptation group, the dyadic maladjustment group characterized by younger age, lower household income, disease duration under 1 year, reduced resilience, and lower financial well-being (such as higher financial toxicity), that can be understood through the intertwined processes of identity disruption, economic shock, and an early post-diagnosis vulnerability window. Chronic illness necessitates reconstructing one’s identity, a challenge especially acute for young and middle-aged adults whose self-concepts and life trajectories remain emergent; disruptions from a cirrhosis diagnosis can therefore destabilize both personal and relational equilibrium (Thomsen et al., 2023; Clur and Barnard, 2024). At the same time, illness-induced work reduction, job loss, and substantial out-of-pocket expenses generate financial toxicity, which is well-documented to impair mental health and family functioning in chronic liver disease (Ufere et al., 2022; Ufere et al., 2022). Furthermore, the first year after diagnosis constitutes a “crisis window” during which adaptation frameworks are nascent and fragile, heightening susceptibility to dyadic maladjustment (Akyirem et al., 2022). These mechanisms underscore the urgency for early, dyad-oriented interventions that integrate narrative or identity-focused therapies to reframe self-concepts, resilience training for both partners, and structured financial navigation (such as charitable support, social aid, and supplementary insurance) to buffer economic burden and foster dyadic adaptation.

The patient-maladjustment profile (low education: elementary school OR = 21.39, junior high OR = 4.3; low resilience OR = 0.84) and the spouse-maladjustment profile (longer disease duration 1–5 years OR = 3.50; Child–Pugh A OR = 0.21; low spouse education: elementary school OR = 19.17, junior high OR = 9.35; absence of spouse chronic disease OR = 0.30; low resilience OR = 0.84; and low family health OR = 0.88) reflect the combined influence of limited health literacy, depleted adaptive capacity, and cumulative caregiving strain. Low formal education plausibly operates through limited health literacy and poorer illness-related knowledge, reducing patients’ ability to understand prognosis, navigate care, and implement self-management, which in turn amplifies psychological distress and maladaptive coping; this mechanism is consistent with recent syntheses linking lower education/health-literacy to worse self-management and psychosocial outcomes across chronic illnesses (Valery and Bernardes, 2021; Gonçalves-Fernández and Pino-Juste, 2025). Psychological resilience emerged as a consistent, independent correlate for both patient-only and spouse-only maladjustment, supporting a resilience-buffer model in which lower individual resources weaken emotion-regulation and problem-solving under chronic stress (Troy et al., 2023); contemporary dyadic studies show that resilience mediates and moderates the relationship between dyadic coping and mental health outcomes, and that resilience-focused education or brief interventions can improve caregiver adaptation (Ren and Ahn, 2025; Tonekaboni et al., 2025). The spouse-maladjustment profile’s association with longer disease duration (1–5 years) and with indicators of greater clinical need (the protective effect of Child-Pugh A implies higher severity raises risk) suggests that cumulative caregiving burden, rather than a single acute event, drives partner distress: longitudinal and cross-sectional literature repeatedly links greater caregiving exposure and escalating patient needs with higher caregiver burden, depression, and poorer family functioning (Virches et al., 2025; van den Kieboom et al., 2020). Notably, the presence of a chronic illness in the spouse increases vulnerability to maladjustment, indicating additive strain when the caregiver also has health limitations; this finding underscores the importance of assessing caregivers’ own health status rather than treating them as an inexhaustible resource (Hong et al., 2016). Finally, reduced family health as a predictor of spouse maladjustment highlights that dyadic outcomes are embedded in family systems: weak family functioning constrains instrumental and emotional supports that otherwise buffer stress, increasing the probability that one partner (often the spouse) may exhibit clinically meaningful maladjustment. Clinically, these patterns recommend a two-fold strategy: (1) early identification of low-education patients and low-resilience dyads (screening for health-literacy, resilience, and caregiver multimorbidity) to triage psychoeducational and resilience-building interventions; and (2) longitudinal caregiver surveillance for couples beyond the first post-diagnosis year, with stepped care (such as skills training, respite and linkage to social/rehabilitative services) for spouses exposed to prolonged caregiving or rising clinical complexity. Together, these targeted, dyad-sensitive approaches address both the upstream (such as education, health-literacy, or resilience) and downstream (such as disease severity and cumulative caregiving) determinants that the multinomial models are identified as key drivers of patient- and spouse-specific maladjustment.

Interestingly, pseudo-synergic adaptation occurred despite milder disease (Child–Pugh A) but was associated with poorer family health, indicating that compensated disease does not guarantee robust family functioning and may coexist with fragile family resources that mask underlying distress. Compensated cirrhosis patients commonly describe persistent uncertainty and impaired role functioning despite “milder” objective indices, which can sustain psychological strain within the household (Hjorth et al., 2020; Hansen et al., 2021). When family health is low—characterized by weaker emotional support, limited healthy routines and constrained resources—dyads are more likely to show superficially coordinated coping (pseudo-synergy) while concealing unresolved anxiety and limited problem-solving capacity; family health predicts self-efficacy and mediates perceived social support in chronic disease populations (Luo et al., 2024). Moreover, caregiver burden and poorer family functioning are seen across the cirrhosis trajectory and are not strictly proportional to disease stage: prolonged caretaking demands, economic strain and role disruption erode family resilience and can produce under-recognized dyadic strain even when patient clinical scores indicate compensation (van den Kieboom et al., 2020; Woodrell et al., 2021). A family-resilience perspective therefore explains why lower objective severity may coexist with worse family health and pseudo-synergic adaptation—the family system’s limited resources and coping flexibility, rather than the patient’s physiology alone, determine true adaptive capacity. Interventions should thus screen family health systematically (not only clinical indices), and prioritize family-level strengthening (such as communication, problem-solving, resource linkage, and targeted psychosocial support) for compensated patients whose families show low resilience, since addressing family functioning may unmask and remediate hidden dyadic distress that clinical severity scores fail to capture (Walsh, 2021).

## 5 Limitation and clinical implications

Several limitations are acknowledged in this study. First, the cross-sectional design precludes causal inferences between resilience, family health, financial toxicity, and dyadic psychosocial adaptation profiles. Longitudinal studies are needed to capture the dynamic interplay and potential bidirectional influences among these factors over the illness trajectory. Second, the single-center design may limit the generalizability of these findings, as cultural, economic, and healthcare system differences across regions could influence dyadic adaptation patterns. Future multi-center studies with geographically diverse samples are warranted. Third, although the study applied a person-centered latent profile analysis to capture heterogeneity, the reliance on self-reported questionnaires may be subject to recall bias, social desirability effects, and unmeasured confounding. Additionally, important psychosocial and relational variables such as marital satisfaction, communication patterns, and social network support were not assessed, which may have provided additional explanatory power for the identified profiles. Fourth, while the study models included multiple patient- and spouse-level predictors, residual confounding from unmeasured clinical or environmental factors cannot be excluded. Furthermore, the latent profile structure was not externally validated through data-splitting or replication in an independent dataset due to the modest sample size (*n* = 321). Future research should seek to replicate these profiles in larger, independent samples to confirm their robustness and generalizability. Fifth, no formal alpha error correction (such as Bonferroni adjustment) was applied to the sequential LMRT and BLRT comparisons during class enumeration. While class decisions in LPA conventionally rely on the convergence of multiple fit indices rather than any single significance test, future methodological studies may explore correction strategies tailored to mixture model class enumeration. Sixth, although Cronbach’s alpha was used to assess internal consistency, future studies may consider reporting McDonald’s omega, which is less dependent on scale length and item-total correlations. Seventh, although questionnaires were checked for completeness on-site and no missing data remained in the final sample, this procedure may have introduced social desirability pressure. Finally, the number and characteristics of dyads who declined participation were not formally recorded, which may limit the assessment of selection bias.

Despite these limitations, the identification of five distinct dyadic psychosocial adaptation profiles underscores the need for precision-oriented, couple-centered interventions in liver cirrhosis care. Screening for key predictors, such as low resilience, poor family health, limited education, and high financial toxicity, may facilitate early risk stratification and tailored intervention planning. For dyads in the maladjustment or pseudo-synergistic profiles, interventions should integrate resilience training, family functioning enhancement, and structured financial counseling. For patient-only or spouse-only maladjustment profiles, targeted psychoeducation addressing health literacy gaps and longitudinal caregiver support programs may mitigate distress and prevent escalation to dyadic maladjustment. Importantly, clinical teams should adopt a dyadic assessment framework, evaluating both patient and spouse adaptation simultaneously rather than focusing solely on the patient. Embedding such couple-sensitive screening and intervention protocols into routine hepatology and psychosocial care could improve not only psychological outcomes but also adherence, disease management, and overall quality of life for both partners.

## 6 Conclusion

This study demonstrates that dyadic psychosocial adaptation among young and middle-aged couples living with liver cirrhosis is highly heterogeneous, with adaptation profiles shaped by the interplay of individual resilience, family health, financial well-being, education, and disease-related factors. Clinical severity alone does not determine psychosocial outcomes; rather, adaptive capacity emerges from the dynamic interaction between personal, relational, and socioeconomic resources. Routine implementation of dyadic, precision-targeted interventions in hepatology care has the potential to alleviate psychosocial distress, strengthen coping capacity, and promote better health and quality-of-life outcomes for both patients and their spouses.

## Data Availability

The original contributions presented in the study are included in the article/Supplementary material, further inquiries can be directed to the corresponding author.
