# Supplementary material for: Latent class analysis of dyadic psychosocial adaptation in young and middle-aged couples with liver cirrhosis
Source: Front Psychol. 2026 Jun 10;17:1798196. doi: 10.3389/fpsyg.2026.1798196 (PMC13290925; doi:10.3389/fpsyg.2026.1798196)
Supplement: Supplementary file 1 [file Supplementary_file_1.docx]

## **Self-Report Psychosocial Adjustment to Illness Scale (PAIS-SR)**

****Instructions:**** The following items ask about your experiences since the onset of your illness. Please choose the response that best describes your situation. There are no right or wrong answers.

| **No.** | **Item** | **Response Options** |
| --- | --- | --- |
| 1 | Which of the following best describes your usual attitude toward taking care of your health? (R) | 0 = I am not very concerned about my health. 1 = Usually I try to pay attention to health matters but sometimes I don‘t. 2 = Most of the time I pay attention to my health needs. 3 = I am very concerned and closely follow my personal health. |
| 2 | In general, how would you describe the quality of medical care available today and the doctors who provide it? (R) | 0 = I have little hope for today’s doctors and medical care. 1 = Medical care and doctors are not as good as they used to be. 2 = The quality of medical care is good, but some areas could be improved. 3 = Medical care could not be better; the doctors do an excellent job. |
| 3 | During your illness, you received services from doctors and other medical staff. How did you feel about the services provided? (R) | 0 = I am very dissatisfied; the staff did not do what they could have done for me. 1 = I am not particularly impressed, but I think they probably did their best. 2 = Despite some problems, the services were good overall. 3 = The services provided by the doctors and staff were excellent. |
| 4 | When people are ill, different people have different expectations and attitudes toward their illness. Please choose the statement that best describes your feelings. (R) | 0 = I feel exhausted and weak due to the illness; sometimes I don‘t know if I can overcome it. 1 = The illness has greatly affected me physically and mentally, but I try hard to overcome it and believe I will return to my former self someday. 2 = The illness has caused me some problems, but I feel I can overcome them fairly quickly. 3 = I am sure I can quickly overcome the illness and its problems and return to my former self. |
| 5 | Being ill can be a confusing experience, and some patients feel that their doctors and medical staff do not provide them with enough information about their condition. Please choose the statement that best describes how you feel in this regard. (R) | 0 = Although I have asked more than once, my doctors and medical staff tell me very little. 1 = I know some things about my condition, but I would like to know more. 2 = I understand my condition quite well, and I can get more information if I wish. 3 = I fully understand my condition; the doctors and staff have provided all the details I want to know. |
| 6 | When faced with an illness such as yours, people often have different thoughts about their treatment and its outcome. Please choose the statement that best describes your expectations of treatment. (R) | 0 = I think the treatment itself is worse than the illness, and I am not sure it is worth accepting. 1 = I dislike certain parts of the treatment; they are unpleasant, but my doctor tells me I must accept them. 2 = I have confidence in my doctor’s guidance regarding treatment, but sometimes I have doubts. 3 = I believe the medical staff are fully capable of guiding my treatment; this is the best treatment I can get. |
| 7 | In managing an illness like yours, patients are told varying amounts of information about their treatment. Please choose the statement that best describes the information you have been told about your treatment. (R) | 0 = I know nothing about my treatment and feel ignored. 1 = I know some information about the treatment, but not as much as I would like. 2 = I know quite a lot about the treatment, though there are still one or two points I would like to know. 3 = I feel fully informed about my treatment and keep up to date. |
| 8 | Does your illness interfere with your ability to work (or study)? If not working or studying, consider daily life activities. | 0 = The illness makes it completely impossible for me to work. 1 = There are some serious problems. 2 = There are only minor problems. 3 = My work has no problem at all. (R) |
| 9 | How would you describe your physical strength at work (or school)? (R) | 0 = Very poor 1 = Not very good 2 = Quite adequate 3 = Very good |
| 10 | In the past 30 days, have you been absent from work (or school) because of your illness? (Consider household tasks or self-care if not applicable.) | 0 = More than 2 weeks 1 = 2 weeks 2 = 1 week 3 = 3 days or fewer (R) |
| 11 | Is work (or school) still as important to you as it was before your illness? (Consider household tasks or self-care if not applicable.) (R) | 0 = It is hardly important to me anymore. 1 = It is far less important than before. 2 = It is slightly less important than before. 3 = It is just as important as before, or even more so. |
| 12 | Have you changed your work (or study) goals because of your illness? (Consider household tasks or self-care if not applicable.) | 0 = I have completely changed my goals. 1 = My goals have changed quite a lot. 2 = My goals have changed slightly. 3 = My goals have not changed. (R) |
| 13 | Since your illness, have you noticed increased problems with colleagues (or classmates, neighbors)? | 0 = Problems have increased a lot. 1 = Problems have increased somewhat. 2 = Problems have slightly increased. 3 = Problems have not increased. (R) |
| 14 | How do you describe the relationship with your husband/wife (or partner) since your illness? (R) | 0 = Very poor 1 = Poor 2 = Fairly good 3 = Very good |
| 15 | How would you describe your relationship with other people at home (e.g., children, parents)? (R) | 0 = Very poor 1 = Poor 2 = Fairly good 3 = Very good |
| 16 | To what extent does your illness interfere with your household responsibilities? | 0 = I have serious difficulties in carrying out household activities. 1 = There are considerable problems, but not all can be overcome. 2 = There are minor problems that are easily overcome. 3 = It does not interfere at all. (R) |
| 17 | Your illness has created difficulties with household tasks. To what extent did family members help you? (R) | 0 = Family members cannot help at all. 1 = Family members try hard to help, but many things remain undone. 2 = Family members do an excellent job, except for a few minor things. 3 = There is no problem at all. |
| 18 | Has your illness led to reduced communication between you and your family members? | 0 = Communication has decreased a lot, and I feel very lonely. 1 = Communication has decreased, and I feel detached from them. 2 = Communication has slightly decreased. 3 = Not at all. (R) |
| 19 | Do you feel that you need help from others with daily tasks? Is that help available? (R) | 0 = I need a lot of help, but no one is around to help. 1 = I sometimes get some help, but not always when I need it. 2 = I need help quite often, and most of the time I receive it. 3 = I do not feel I need help, or I can get help from family or friends when needed. |
| 20 | Illness such as yours can sometimes cause a great financial burden on the family. Do you have financial difficulties in seeking medical care? (R) | 0 = Severe financial difficulties 1 = Moderate financial difficulties 2 = Slight financial difficulties 3 = No financial difficulties |
| 21 | Has your illness caused changes in your relationship with your husband/wife (or partner)? | 0 = We have serious problems because of the illness, or the relationship has broken down. 1 = We have been noticeably less close since the illness. 2 = We are slightly less close than before the illness. 3 = Our relationship has not changed at all. (R) |
| 22 | Have you had arguments with your partner because of your illness? | 0 = We argue all the time. 1 = We argue frequently. 2 = We argue occasionally. 3 = We have no arguments. (R) |
| 23 | Since your illness, has your desire for sexual activity decreased? | 0 = I have no desire for sexual activity at all. 1 = My desire has clearly decreased. 2 = My desire has slightly decreased. 3 = My desire has not decreased. (R) |
| 24 | Has your illness led to a decrease in the frequency of sexual activity? | 0 = I have stopped having sex. 1 = There has been a clear decrease. 2 = There has been a slight decrease. 3 = There has been no decrease. (R) |
| 25 | Has the pleasure or satisfaction you experience from sexual activity changed? | 0 = Sexual satisfaction and pleasure are completely lost. 1 = Sexual satisfaction and pleasure have clearly decreased. 2 = Sexual satisfaction and pleasure have slightly decreased. 3 = Sexual satisfaction has not decreased. (R) |
| 26 | How has your illness affected your ability to engage in sexual activity? | 0 = I am completely unable to have sexual activity. 1 = There are always problems with sexual activity. 2 = Sexual activity is slightly affected. 3 = My sexual ability has not been affected. (R) |
| 27 | Sometimes illness affects the normal sexual relationship between partners, leading to arguments or problems. Have you and your partner had such arguments? | 0 = We argue all the time. 1 = We argue frequently. 2 = We argue occasionally. 3 = We have no arguments. (R) |
| 28 | Has there been any change in the frequency of sexual activity since your illness? | 0 = Stopped sexual activity entirely 1 = Clearly decreased 2 = Slightly decreased 3 = No decrease (R) |
| 29 | Since your illness, have you maintained contact with family members outside your home (by phone or in person) as before? (R) | 0 = No contact 1 = Contact has clearly decreased. 2 = Contact has slightly decreased. 3 = Contact is the same as before, or even more frequent. |
| 30 | Since your illness, do you still want to spend time with family members? (R) | 0 = I have no interest in this. 1 = My interest is clearly less than before. 2 = My interest is slightly less. 3 = My interest is the same as before, or even greater. |
| 31 | After getting ill, people sometimes have to ask family members outside the home for material help. Have you needed this? Did they give you what you needed? (R) | 0 = Although I need a lot, they help very little. 1 = They gave me some help, but not enough. 2 = They gave me sufficient help, except for minor things. 3 = I do not need help, or they gave me everything I needed. |
| 32 | Sometimes people interact a great deal with family members outside the immediate family. Do you interact much with these extended family members, and has your illness reduced this contact? | 0 = Contact has been almost completely abandoned. 1 = Contact has clearly decreased. 2 = Contact has decreased to some extent. 3 = Contact has not been affected, or I do not have such interactions. (R) |
| 33 | Overall, how have you been getting along with these family members lately? (R) | 0 = Very poor 1 = Poor 2 = Fairly good 3 = Very good |
| 34 | Are you still as interested in your hobbies and leisure activities as you were before your illness? (R) | 0 = I am not interested at all. 1 = My interest is clearly less than before. 2 = My interest is slightly less than before. 3 = My interest is the same as before. |
| 35 | How about your actual participation? Are you still taking part in these activities? (R) | 0 = Now I barely participate. 1 = My participation has clearly decreased. 2 = My participation has slightly decreased. 3 = My participation has not decreased. |
| 36 | Is your interest in family recreational activities (e.g., playing cards, games, traveling, swimming) the same as before your illness? (R) | 0 = I am not interested at all. 1 = My interest is clearly less than before. 2 = My interest is slightly less than before. 3 = My interest is the same as before. |
| 37 | Do you still participate in family recreational activities as you did in the past? (R) | 0 = Now I barely participate. 1 = My participation has clearly decreased. 2 = My participation has slightly decreased. 3 = My participation has not decreased. |
| 38 | Is your interest in social activities (e.g., social clubs, movies) the same as before your illness? (R) | 0 = I am not interested at all. 1 = My interest is clearly less than before. 2 = My interest is slightly less than before. 3 = My interest is the same as before. |
| 39 | How about your actual participation? Do you still go out socializing with friends? (R) | 0 = Now I barely participate. 1 = My participation has clearly decreased. 2 = My participation has slightly decreased. 3 = My participation has not decreased. |
| 40 | Recently, have you felt afraid, tense, or anxious? | 0 = Not at all (R) 1 = Occasionally 2 = Sometimes 3 = Often |
| 41 | Recently, have you felt sad, depressed, uninterested in things, or hopeless? | 0 = Often 1 = Sometimes 2 = Occasionally 3 = Not at all (R) |
| 42 | Recently, have you felt angry, irritable, and had difficulty controlling your temper? | 0 = Not at all (R) 1 = Occasionally 2 = Sometimes 3 = Often |
| 43 | Recently, have you felt self-blame, guilt, or that you have let others down? | 0 = Often 1 = Sometimes 2 = Occasionally 3 = Not at all (R) |
| 44 | Recently, have you worried about your illness or other matters? | 0 = Not at all (R) 1 = Occasionally 2 = Sometimes 3 = Often |

****Scoring Note:**** Items marked with “(R)” indicate that higher raw scores reflect better adjustment; these items are reverse-coded before calculating domain and total scores so that higher scores uniformly indicate poorer adjustment across all items.

## **Self-Report Psychosocial Adjustment to Illness Scale – Caregiver Version (PAIS-SR)**

****Instructions to the spouse:**** As the primary caregiver for your husband/wife, please answer the following questions based on your own experiences and feelings since your partner's illness. There are no right or wrong answers.

| **No.** | **Item** | **Response Options** |
| --- | --- | --- |
| 1 | Which of the following best describes your usual attitude toward taking care of your own health as a caregiver? (R) | 0 = I am not very concerned about my health. 1 = Usually I try to pay attention to health matters but sometimes I don’t. 2 = Most of the time I pay attention to my health needs. 3 = I am very concerned and closely follow my personal health. |
| 2 | In general, how would you describe the quality of medical care available today and the doctors who provide it? (R) | 0 = I have little hope for today’s doctors and medical care. 1 = Medical care and doctors are not as good as they used to be. 2 = The quality of medical care is good, but some areas could be improved. 3 = Medical care could not be better; the doctors do an excellent job. |
| 3 | During your spouse’s illness, you received services from doctors and other medical staff. How did you feel about the services provided to you as a caregiver? (R) | 0 = I am very dissatisfied; the staff did not do what they could have done for me. 1 = I am not particularly impressed, but I think they probably did their best. 2 = Despite some problems, the services were good overall. 3 = The services provided by the doctors and staff were excellent. |
| 4 | When people are in a caregiving role, different people have different expectations and attitudes. Please choose the statement that best describes your feelings about your situation. (R) | 0 = I feel exhausted and weak due to caregiving; sometimes I don’t know if I can overcome it. 1 = Caregiving has greatly affected me physically and mentally, but I try hard to overcome it and believe I will return to my former self someday. 2 = Caregiving has caused me some problems, but I feel I can overcome them fairly quickly. 3 = I am sure I can quickly overcome the caregiving-related problems and return to my former self. |
| 5 | Being a caregiver can be a confusing experience, and some feel that doctors and medical staff do not provide them with enough information. Please choose the statement that best describes how you feel in this regard. (R) | 0 = Although I have asked more than once, my doctors and medical staff tell me very little. 1 = I know some things about my partner's condition, but I would like to know more. 2 = I understand my partner's condition quite well, and I can get more information if I wish. 3 = I fully understand my partner's condition; the doctors and staff have provided all the details I want to know. |
| 6 | When faced with your spouse's illness, people often have different thoughts about the treatment and its outcome. Please choose the statement that best describes your expectations. (R) | 0 = I think the treatment itself is worse than the illness, and I am not sure it is worth accepting. 1 = I dislike certain parts of the treatment; they are unpleasant, but the doctor tells us we must accept them. 2 = I have confidence in the doctor’s guidance regarding treatment, but sometimes I have doubts. 3 = I believe the medical staff are fully capable of guiding treatment; this is the best treatment my spouse can get. |
| 7 | In managing an illness like your spouse's, caregivers are told varying amounts of information. Please choose the statement that best describes the information you have been given. (R) | 0 = I know nothing about the treatment and feel ignored. 1 = I know some information about the treatment, but not as much as I would like. 2 = I know quite a lot about the treatment, though there are still one or two points I would like to know. 3 = I feel fully informed about the treatment and keep up to date. |
| 8 | Does your role as a caregiver interfere with your ability to work (or study)? If not working or studying, consider daily life activities. | 0 = The caregiving makes it completely impossible for me to work. 1 = There are some serious problems. 2 = There are only minor problems. 3 = My work has no problem at all. (R) |
| 9 | How would you describe your physical strength at work (or school) since becoming a caregiver? (R) | 0 = Very poor 1 = Not very good 2 = Quite adequate 3 = Very good |
| 10 | In the past 30 days, have you been absent from work (or school) because of your caregiving responsibilities? | 0 = More than 2 weeks 1 = 2 weeks 2 = 1 week 3 = 3 days or fewer (R) |
| 11 | Is work (or school) still as important to you as it was before you became a caregiver? (R) | 0 = It is hardly important to me anymore. 1 = It is far less important than before. 2 = It is slightly less important than before. 3 = It is just as important as before, or even more so. |
| 12 | Have you changed your work (or study) goals because of your caregiving role? | 0 = I have completely changed my goals. 1 = My goals have changed quite a lot. 2 = My goals have changed slightly. 3 = My goals have not changed. (R) |
| 13 | Since becoming a caregiver, have you noticed increased problems with colleagues (or classmates, neighbors)? | 0 = Problems have increased a lot. 1 = Problems have increased somewhat. 2 = Problems have slightly increased. 3 = Problems have not increased. (R) |
| 14 | How do you describe the relationship with your husband/wife (the patient) since the illness? (R) | 0 = Very poor 1 = Poor 2 = Fairly good 3 = Very good |
| 15 | How would you describe your relationship with other people at home (e.g., children, parents) since your spouse's illness? (R) | 0 = Very poor 1 = Poor 2 = Fairly good 3 = Very good |
| 16 | To what extent does caregiving interfere with your household responsibilities? | 0 = I have serious difficulties in carrying out household activities. 1 = There are considerable problems. 2 = There are minor problems that are easily overcome. 3 = It does not interfere at all. (R) |
| 17 | Your spouse's illness has created difficulties with household tasks. To what extent did other family members help you? (R) | 0 = Family members cannot help at all. 1 = Family members try hard to help, but many things remain undone. 2 = Family members do an excellent job, except for a few minor things. 3 = There is no problem at all. |
| 18 | Has your role as a caregiver led to reduced communication between you and your family members? | 0 = Communication has decreased a lot, and I feel very lonely. 1 = Communication has decreased, and I feel detached from them. 2 = Communication has slightly decreased. 3 = Not at all. (R) |
| 19 | Do you feel that you need help from others with daily tasks as a caregiver? Is that help available? (R) | 0 = I need a lot of help, but no one is around to help. 1 = I sometimes get some help, but not always when I need it. 2 = I need help quite often, and most of the time I receive it. 3 = I do not feel I need help, or I can get help from family or friends when needed. |
| 20 | Illness such as your spouse's can sometimes cause a great financial burden on the family. Do you have financial difficulties because of the illness? (R) | 0 = Severe financial difficulties 1 = Moderate financial difficulties 2 = Slight financial difficulties 3 = No financial difficulties |
| 21 | Has your spouse's illness caused changes in your relationship with him/her? | 0 = We have serious problems because of the illness, or the relationship has broken down. 1 = We have been noticeably less close since the illness. 2 = We are slightly less close than before the illness. 3 = Our relationship has not changed at all. (R) |
| 22 | Have you had arguments with your spouse because of the illness or caregiving? | 0 = We argue all the time. 1 = We argue frequently. 2 = We argue occasionally. 3 = We have no arguments. (R) |
| 23 | Since your spouse's illness, has your own desire for sexual activity decreased? | 0 = I have no desire for sexual activity at all. 1 = My desire has clearly decreased. 2 = My desire has slightly decreased. 3 = My desire has not decreased. (R) |
| 24 | Has your spouse's illness led to a decrease in the frequency of sexual activity in your relationship? | 0 = We have stopped having sex. 1 = There has been a clear decrease. 2 = There has been a slight decrease. 3 = There has been no decrease. (R) |
| 25 | Has the pleasure or satisfaction you experience from sexual activity changed? | 0 = Sexual satisfaction and pleasure are completely lost. 1 = Sexual satisfaction and pleasure have clearly decreased. 2 = Sexual satisfaction and pleasure have slightly decreased. 3 = Sexual satisfaction has not decreased. (R) |
| 26 | How has your spouse's illness affected your own ability to engage in sexual activity? | 0 = I am completely unable to have sexual activity. 1 = There are always problems with sexual activity. 2 = Sexual activity is slightly affected. 3 = My sexual ability has not been affected. (R) |
| 27 | Sometimes illness affects the normal sexual relationship between partners, leading to arguments. Have you and your spouse had such arguments? | 0 = We argue all the time. 1 = We argue frequently. 2 = We argue occasionally. 3 = We have no arguments. (R) |
| 28 | Has there been any change in the frequency of sexual activity since your spouse's illness? | 0 = Stopped sexual activity entirely 1 = Clearly decreased 2 = Slightly decreased 3 = No decrease (R) |
| 29 | Since your spouse's illness, have you maintained contact with family members outside your home (by phone or in person) as before? (R) | 0 = No contact 1 = Contact has clearly decreased. 2 = Contact has slightly decreased. 3 = Contact is the same as before, or even more frequent. |
| 30 | Since becoming a caregiver, do you still want to spend time with extended family members? (R) | 0 = I have no interest in this. 1 = My interest is clearly less than before. 2 = My interest is slightly less. 3 = My interest is the same as before, or even greater. |
| 31 | As a caregiver, have you needed material help from family members outside your home? Did they give you what you needed? (R) | 0 = Although I need a lot, they help very little. 1 = They gave me some help, but not enough. 2 = They gave me sufficient help, except for minor things. 3 = I do not need help, or they gave me everything I needed. |
| 32 | Do you interact much with extended family members, and has your caregiving role reduced this contact? | 0 = Contact has been almost completely abandoned. 1 = Contact has clearly decreased. 2 = Contact has decreased to some extent. 3 = Contact has not been affected, or I do not have such interactions. (R) |
| 33 | Overall, how have you been getting along with extended family members lately? (R) | 0 = Very poor 1 = Poor 2 = Fairly good 3 = Very good |
| 34 | Are you still as interested in your hobbies and leisure activities as you were before becoming a caregiver? (R) | 0 = I am not interested at all. 1 = My interest is clearly less than before. 2 = My interest is slightly less than before. 3 = My interest is the same as before. |
| 35 | How about your actual participation? Are you still taking part in these activities? (R) | 0 = Now I barely participate. 1 = My participation has clearly decreased. 2 = My participation has slightly decreased. 3 = My participation has not decreased. |
| 36 | Is your interest in family recreational activities (e.g., playing cards, games, traveling) the same as before your spouse's illness? (R) | 0 = I am not interested at all. 1 = My interest is clearly less than before. 2 = My interest is slightly less than before. 3 = My interest is the same as before. |
| 37 | Do you still participate in family recreational activities as you did in the past? (R) | 0 = Now I barely participate. 1 = My participation has clearly decreased. 2 = My participation has slightly decreased. 3 = My participation has not decreased. |
| 38 | Is your interest in social activities (e.g., social clubs, movies) the same as before? (R) | 0 = I am not interested at all. 1 = My interest is clearly less than before. 2 = My interest is slightly less than before. 3 = My interest is the same as before. |
| 39 | How about your actual participation? Do you still go out socializing with friends? (R) | 0 = Now I barely participate. 1 = My participation has clearly decreased. 2 = My participation has slightly decreased. 3 = My participation has not decreased. |
| 40 | Recently, have you felt afraid, tense, or anxious? | 0 = Not at all (R) 1 = Occasionally 2 = Sometimes 3 = Often |
| 41 | Recently, have you felt sad, depressed, uninterested in things, or hopeless? | 0 = Often 1 = Sometimes 2 = Occasionally 3 = Not at all (R) |
| 42 | Recently, have you felt angry, irritable, and had difficulty controlling your temper? | 0 = Not at all (R) 1 = Occasionally 2 = Sometimes 3 = Often |
| 43 | Recently, have you felt self-blame, guilt, or that you have let others down? | 0 = Often 1 = Sometimes 2 = Occasionally 3 = Not at all (R) |
| 44 | Recently, have you worried about your spouse's illness or other matters? | 0 = Not at all (R) 1 = Occasionally 2 = Sometimes 3 = Often |

****Scoring Note:**** Items marked with “(R)” are reverse-coded before calculating domain and total scores, so that higher scores uniformly indicate poorer psychosocial adjustment. As in the patient version, scale direction alternates on every other item.

## **Resilience Scale (CD-RISC-10)**

****Instructions:**** Please indicate how much you agree with the following statements as they apply to you over the past month.

| No. | Item | Not true at all (0) | Rarely true (1) | Sometimes true (2) | Often true (3) | True nearly always (4) |
| --- | --- | --- | --- | --- | --- | --- |
| 1 | I am able to adapt when changes occur. | □ | □ | □ | □ | □ |
| 2 | I can deal with whatever comes my way. | □ | □ | □ | □ | □ |
| 3 | I try to see the humorous side of things when I am faced with problems. | □ | □ | □ | □ | □ |
| 4 | Having to cope with stress can make me stronger. | □ | □ | □ | □ | □ |
| 5 | I tend to bounce back after illness, injury, or other hardships. | □ | □ | □ | □ | □ |
| 6 | I believe I can achieve my goals, even if there are obstacles. | □ | □ | □ | □ | □ |
| 7 | Under pressure, I stay focused and think clearly. | □ | □ | □ | □ | □ |
| 8 | I am not easily discouraged by failure. | □ | □ | □ | □ | □ |
| 9 | I think of myself as a strong person when dealing with life's challenges and difficulties. | □ | □ | □ | □ | □ |
| 10 | I am able to handle unpleasant or painful feelings like sadness, fear, and anger. | □ | □ | □ | □ | □ |

****Family Health Scale – Short Form (FHS-SF)****

****Instructions:**** Please indicate your level of agreement with the following statements about your family.

| **No.** | **Item** | **Strongly Disagree**  **(1)** | **Somewhat Disagree**  **(2)** | **Neither Agree nor Disagree (3)** | **Somewhat Agree**  **(4)** | **Strongly Agree**  **(5)** |
| --- | --- | --- | --- | --- | --- | --- |
| 1 | In my family, we support each other. | □ | □ | □ | □ | □ |
| 2 | In my family, I feel secure in my family relationships. | □ | □ | □ | □ | □ |
| 3 | In my family, we maintain hope even during difficult times. | □ | □ | □ | □ | □ |
| 4 | In my family, we help each other seek healthcare services when needed (e.g., making appointments). | □ | □ | □ | □ | □ |
| 5 | In my family, we help each other make changes for better health. | □ | □ | □ | □ | □ |
| 6 | In my family, we do not trust healthcare professionals. (R) | □ | □ | □ | □ | □ |
| 7 | In the past 12 months, after paying for basic living expenses, our family had no spare money. (R) | □ | □ | □ | □ | □ |
| 8 | In the past 12 months, my family's housing did not meet our family's needs. (R) | □ | □ | □ | □ | □ |
| 9 | When we encounter problems at school or work, our family can seek help from people outside our family. | □ | □ | □ | □ | □ |
| 10 | If we need financial assistance, our family can borrow money from people outside our family (e.g., 1,000 RMB). | □ | □ | □ | □ | □ |

****Scoring Note:**** Items are rated on a 5-point scale. Items marked with (R) in the ‘Family Health Resources’ dimension are reverse-coded.

## **Financial Toxicity Scale (COST-PROM)**

****Instructions:**** Please indicate how much each statement applies to you over the past 7 days. There are no right or wrong answers.

| **No.** | **Item** | **Not at all**  **(0)** | **A little bit**  **(1)** | **Somewhat**  **(2)** | **Quite a bit**  **(3)** | **Very much**  **(4)** |
| --- | --- | --- | --- | --- | --- | --- |
| 1 | I know that I have enough money in savings, retirement, or assets to cover the costs of my treatment. (reverse-scored) | □ | □ | □ | □ | □ |
| 2 | My out-of-pocket medical expenses are more than I thought they would be. | □ | □ | □ | □ | □ |
| 3 | I worry about the financial problems I will have in the future as a result of my illness or treatment. | □ | □ | □ | □ | □ |
| 4 | I feel I have no choice about the amount of money I spend on care. | □ | □ | □ | □ | □ |
| 5 | I am frustrated that I cannot work or contribute as much as I usually do. | □ | □ | □ | □ | □ |
| 6 | I am satisfied with my current financial situation. (reverse-scored) | □ | □ | □ | □ | □ |
| 7 | I am able to meet my monthly expenses. (reverse-scored) | □ | □ | □ | □ | □ |
| 8 | I feel financially stressed. | □ | □ | □ | □ | □ |
| 9 | I am concerned about keeping my job and income, including work at home. | □ | □ | □ | □ | □ |
| 10 | My illness or treatment has reduced my satisfaction with my present financial situation. | □ | □ | □ | □ | □ |
| 11 | I feel in control of my financial situation. (reverse-scored) | □ | □ | □ | □ | □ |
